# To be or not to be phosphorylated: understanding the role of Ebola virus nucleoprotein in the dynamic interplay with the transcriptional activator VP30 and the host phosphatase PP2A-B56

**DOI:** 10.1080/22221751.2024.2447612

**Published:** 2024-12-27

**Authors:** Lennart Kämper, Ida Kuhl, Melina Vallbracht, Thomas Hoenen, Uwe Linne, Axel Weber, Petr Chlanda, Michael Kracht, Nadine Biedenkopf

**Affiliations:** a Institute of Virology, Philipps-Universität Marburg, Marburg, Germany; b Department of Infectious Diseases, Virology, Heidelberg University, Heidelberg, Germany; c Research Center for Quantitative Analysis of Molecular and Cellular Systems – BioQuant, Heidelberg University, Heidelberg, Germany; d Institute of Molecular Virology and Cell Biology, Friedrich-Loeffler-Institut, Greifswald, Germany; e Mass Spectrometry Facility, Department of Chemistry, Philipps-Universität Marburg, Marburg, Germany; f Rudolf Buchheim Institute of Pharmacology, Justus-Liebig University Gießen, Gießen, Germany

**Keywords:** Ebola virus, nucleoprotein, VP30, host phosphatase PP2A, viral transcription, phosphorylation, regulation

## Abstract

Ebola virus (EBOV) transcription is essentially regulated via dynamic dephosphorylation of its viral transcription activator VP30 by the host phosphatase PP2A. The nucleoprotein NP has emerged as a third key player in the regulation of this process by recruiting both the regulatory subunit B56 of PP2A and its substrate VP30 to initiate VP30 dephosphorylation and hence viral transcription. Both binding sites are located in close proximity to each other in NP’s C-terminal-disordered region. This study investigates NP’s role in VP30 dephosphorylation and transcription activation, focussing on the spatial requirements of NP’s binding sites. Increasing the distance between PP2A-B56 and VP30 at the NP interface revealed that close spatial and orientational contact is necessary for efficient VP30 dephosphorylation and viral transcription. Longer distances were lethal for recombinant EBOV except when a compensatory mutation, NP-T603I, occurred. This mutation, located between the NP binding sites for PP2A-B56 and VP30, fully restored functionality. Mass spectrometry showed that T603 is phosphorylated in recEBOV-NPwt virions. Mutational analysis indicated that T603I facilitates VP30 dephosphorylation in otherwise lethal recEBOV and that dynamic phosphorylation of NP-T603 is important for efficient primary viral transcription in the WT context. These findings emphasize the critical and evolutionarily pressured interplay between VP30 and PP2A-B56 within the NP C-terminal-disordered region and highlight the important role of NP on the regulation of viral transcription during the EBOV life cycle.

## Introduction

Ebola virus (EBOV) (genus *Orthoebolavirus,* family *Filoviridae*) is among the most lethal viruses known, averaging a lethality of 40–60% [1]. Since its first appearance in the Democratic Republic of the Kongo (former Zaire) in 1976, EBOV outbreaks have sporadically occurred in central and western African countries, among them the West African EBOV outbreak 2014–2016 with more than 11,000 fatalities [2]. EBOV infections cause severe febrile Ebola virus disease, accompanied by fatigue, gastrointestinal disorders, rash and frequently haemorrhages [3]. Understanding the molecular mechanisms of EBOV replication and how the virus exploits host cell factors for an efficient viral life cycle is indispensable for further development of antivirals that might have broad-spectrum antiviral activity against different *Orthoebolavirus* species.

EBOV has a non-segmented, negative-strand RNA genome of 19 kb and codes for seven structural proteins: the nucleoprotein (NP), the viral protein (VP) 35, VP40, glycoprotein (GP), VP30, VP24, and the RNA-dependent RNA polymerase (L) [4–8]. The enveloped virus contains GP as the sole surface protein, mediating the infection of target cells [9]. The matrix protein VP40 is involved in the budding of novel viral particles, while VP24 plays a role in nucleocapsid condensation and subsequent trafficking to the budding sites at the plasma membrane [10–13]. The single-stranded RNA (both genomic and antigenomic) is encapsidated by NP, which is essential for viral RNA synthesis by the other nucleocapsid proteins: the polymerase L, its cofactor VP35 and the transcriptional activator VP30. While NP, VP35, and L are sufficient to facilitate viral replication, transcription of monocistronic viral mRNAs is additionally depending on VP30 [7, 14, 15].

**Figure 1.**
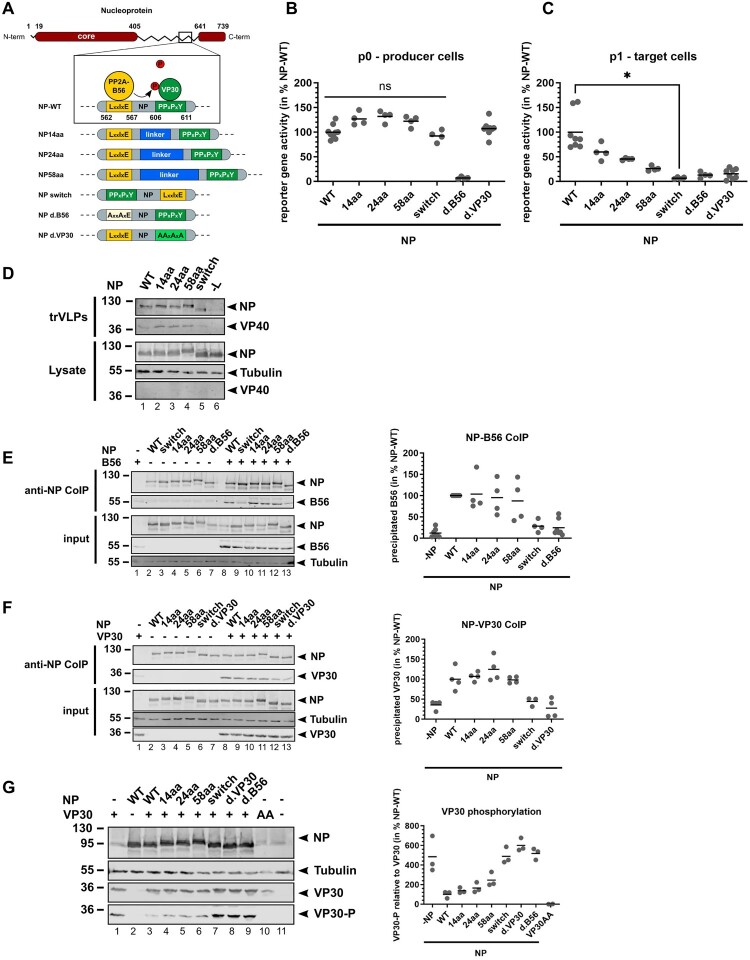
Transcription activation requires a close spatial and orientational interplay between NP, PP2A-B56α, and VP30. (A) EBOV NP mutants carrying mutations at the PP2A-B56α (orange) and VP30 (green) binding interfaces that were utilized for the following experiments. (B) Transcription and replication competent virus-like particle (trVLP) assay with a tetracistronic minigenome, reporter gene activity in producer cells (p0). 72 hours post transfection (hpt), luciferase activity was measured reflecting EBOV-specific transcription. NPwt set to 100%. Mean of individual experiments, ns: not statistically significant. (C) Reporter gene activity in infected target cells (p1). Generated trVLPs from (B) supernatants were collected and concentrated via ultracentrifugation to infect naïve p1 cells. Luciferase activity was measured 72 hpt reflecting primary transcription. NPwt set to 100%. Mean of individual experiments. (D) Expression of NP and VP40 in p0 cell lysates and incorporation into trVLPs analysed by WB analysis. Samples generated as described in panels B and C. (E–F) Co-immunoprecipitation of NP mutants with B56α (E) or VP30 (F) in HEK293 cells using anti-flag antibody covered magnetic beads. Flag-tagged NP is precipitated together with potential binding partners that are subsequently detected through WB. On the right side, quantification from individual experiments. NPwt set to 100%. (G) VP30 phosphorylation in the presence of NP upon recombinant expression in HeLa cells. VP30AA was included as positive control for dephosphorylated VP30 (lane 11 [20]). On the right side, quantification from individual experiments. NPwt set to 100%.

VP30 is dynamically phosphorylated in its N-terminal domain with serine 29 phosphorylation crucial for its regulatory function [14–19]. Phosphorylation impacts VP30’s interaction with viral RNA and NP, influencing transcription initiation and packaging into new virions [19, 20]. Reversible VP30 phosphorylation by host kinase/s and phosphatase/s contributes essentially to EBOV propagation. While phosphorylation of VP30 is enabled by the cellular serine/arginine-rich protein kinase 1 (SRPK1) [21] as well as LATS 1 and 2 [22], dephosphorylation of VP30 and hence activation of viral transcription is mediated by the cellular protein phosphatases 1 (PP1) and 2A (PP2A) [15, 18]. We could recently determine the underlying mechanism of how EBOV and also Marburg virus VP30 are dephosphorylated by PP2A: the regulatory subunit B56α of PP2A as well as its substrate VP30 directly bind to NP, that thereby acts as a scaffold protein bringing both proteins in close contact, a mechanism that is conserved among filoviruses [23, 24]. As VP30 dephosphorylation by PP2A is crucial for the activation of viral transcription, NP has emerged as the third key factor in the regulation of this process. Both binding sites are located within the NP C-terminal internal-disordered region (IDR) being important for the inclusion body formation and interaction with viral and host proteins [25–27] (Figure 1A). Interestingly, both binding sites, PP2A-B56α (LxxIxE, amino acid (aa) 562–567) and VP30 (PPxPxY motif, aa 606–611), are in close proximity and highly conserved among filoviral NPs [23, 24, 28].

In the following study, we aimed to comprehend the role of EBOV NP for the regulation of VP30 dephosphorylation and hence transcription activation by investigating the minimal spatial requirements of the two binding sites on NP. The introduction of a rigid amino acid linker to increase the distance between PP2A-B56α and VP30 revealed that PP2A-B56α and VP30 need to be in close spatial and orientational contact within the NP molecule to facilitate efficient VP30 dephosphorylation and subsequent viral transcription. The insertion of a longer distance was lethal for the generation of recombinant (rec)EBOV except when eventually a single compensatory point mutation occurred in NP. Intriguingly, the compensatory mutation T603I was located between the NP binding sites for PP2A-B56α and VP30 and could completely restore the insertion-induced defects in recEBOV. Mass spectrometry analyses revealed that this threonine residue at NP position 603 is phosphorylated in recEBOV-NPwt virions. Mutational analysis towards phosphomimetic NP-T603 mutants showed that the compensatory mutation T603I increased VP30 dephosphorylation in the otherwise lethal recombinant EBOV with an increased PP2A-VP30 distance. Our data further suggest that dynamic phosphorylation of NPwt-T603 is essential during the activation of primary viral transcription.

Together, our data emphasize the spatial interplay between VP30 and PP2A-B56α within the NP IDR as an essential and highly regulated process that underlies a strong evolutionary pressure. The importance of NP dynamic phosphorylation at position T603 represents a further mechanism in this interplay, contributing to efficient primary viral transcription independent of VP30 dephosphorylation. Together, this proposes the NP C-terminal IDR as a highly dynamic and flexible platform that allows exerting the multifaceted functions of NP and interactions with viral and host proteins during the EBOV life cycle.

## Materials and methods

### Cell culture

HEK293F (embryonic human kidney), HuH7 (human hepatoma), Vero E6 (African green monkey kidney), and HeLa (Human cervical cancer) cells were cultivated in Dulbecco’s modified Eagle Medium (DMEM) supplemented with 10% fetal bovine calf serum (FCS) and 5 mM glutamine as well as penicillin/streptomycin and incubated at 37°C, 5% CO_2_.

### Plasmids

pCAGGS plasmids encoding for EBOV wt proteins, T7 polymerase, pGL4 Firefly, and minigenomes (MG) were described before [10, 12]. Flag-tagged VP30, VP30AA, as well as NPd.VP30 and NPd.B56 mutants were described here [15, 16, 23]. pCAGGS-PP2A-B56α-myc was subcloned from pcDNA5-FRT-TO-YFP-B56α [23] into pCAGGS. For cloning of NP mutants with an increased distance between PP2A-B56α and VP30 (NP14aa, NP24aa, and NP58aa), a rigid linker (EAAAK)n was subcloned together with flanking restriction enzyme sites into pCAGGS NPflag plasmid. pCAGGS NPswitch as well as the pCAGGS NP-T603I and NP-T603D were cloned using the QuikChange lightning multi-site-directed mutagenesis kit (Agilent Technologies) according to the manufacturer’s instructions. cDNA for recEBOV-NP mutants was cloned by subcloning into a full-length pAMP EBOV Mayinga plasmid using restriction enzymes [19]. All sequences were verified by sequencing. A detailed cloning strategy and primer sequences are available on request.

### Co-immunoprecipitation

Transfection and lysis of cells were performed as described in reference [16]. Precipitation of flag-tagged NP in combination with PP2A-B56 was performed using mouse anti-flag M2 agarose (Sigma-Aldrich, A2220) for 2 h rotating at 4°C. Precipitation of flag-tagged NP in combination with VP30 was performed using mouse anti-flag magnetic beads (MedChemExpress HY-K0207) according to the manufacturer’s recommendations for 10 minutes at room temperature. For further details, see Supplemental methods.

### Electrophoresis and western blot

SDS-PAGE and WBs were performed as described in reference [29]. For further details and used antibodies, see Supplemental methods.

### Ebola virus-specific transcription and replication competent virus-like particle assay

Transcription and replication competent virus-like particle (trVLP) assay using a tetracistronic minigenome coding for a nano-luciferase reporter, VP40, GP, and VP24 was performed as previously described [12, 30]. trVLP assay using a monocistronic minigenome with the individual expression of VP40, GP, and VP24 was performed as described in ref. [31]. Luciferase activity in p0 cells was measured at 72hpt. trVLPs were purified from p0 supernatants via ultracentrifugation and used for infection of p1 cells or for SDS-PAGE and WB. Luciferase activity in infected p1 cells was measured 72 h post-infection. For further details, see Supplemental methods.

### Rescue of recombinant Ebola virus

All experiments with recombinant (rec) EBOV were conducted under BSL4 conditions at the Philipps-University Marburg according to national regulations. Rescue of recEBOV was performed as described in ref. [19]. For further details, see Supplemental methods.

### Growth kinetics of recombinant Ebola virus

VeroE6 cells were infected with recEBOVs with a multiplicity of infection (MOI) of 0.1 or 0.01 for 1 h at 37°C and 5% CO_2._ Samples from supernatants were taken from 0 to 7 dpi as indicated. RNA from cell culture supernatant was collected at day 7 pi for sequencing. Infectious virus from supernatants was analysed in quadruplicates by TCID_50_ analysis in VeroE6 cells. For further details, see Supplemental methods.

### Detection of phosphorylated VP30 by western blot analysis

Phosphorylation state of VP30 was measured upon recombinant expression of VP30 and NP in HeLa cells [23]. At 48 hpt, cells were lysed and subjected to WB analysis. Phosphorylated VP30 at position 29 was detected by a phospho-specific antibody (anti pS29 VP30, rabbit) [20].

### RT-qPCR of Ebola virus vRNA

Cell culture supernatants from recEBOV-infected HuH7 cells containing 1.2 × 10^5^ PFU were subjected to RNA isolation using the QIAmp Viral RNA mini kit from Qiagen following the manufacturer’s instructions (ref. 52906). RT-qPCRs of viral RNA were performed as previously described [32]. For further details, see Supplemental methods.

### Indirect immunofluorescence analysis

Indirect immunofluorescence analysis was performed in HuH7 cells as described earlier [29]. Cells were infected with recEBOV (MOI 0.3), fixed with 4% PFA at 24 h post-infection (hpi), permeabilized, and stained with protein-specific antibodies for NP and VP40. Images were taken using a Stellaris 8 confocal scanning laser microscope from Leica with a 63x objective. Images were processed with ImageJ/Fiji. For further details and antibodies, see Supplemental material.

### Cryo-electron tomography (cryo-ET)

Supernatants from HuH7 cells infected with the respective recEBOVs were purified and fixed at day 3 (recEBOV-NPwt and recEBOV-NP58aa-T603I) or day 7 pi (attenuated recEBOV-NP14aa). Samples were vitrified using a GP2 plunge freezer (Leica) and analysed in a Titan Krios Transmission Electron Microscope (ThermoFisher Scientific). For visualization, 10 slices of the final tomogram were averaged. For further details, see Supplemental methods.

### Mass spectrometry

Wildtype recEBOV, recEBOV-NP-14aa, and recEBOV-NP-58aa-T603I were purified from HuH7 as described for cryo-EM analysis. Samples were inactivated by boiling at 100 °C in 1% SDS and subjected to mass spectrometry analysis. For a detailed description, see Supplemental methods.

### Statistics

For statistical analysis, a one-way ANOVA with multiple comparisons was performed using GraphPad Prism 10.0.2 (ns *p* > 0.05, *p ≤ 0.05, **p ≤ 0.01, ***p ≤ 0.001, ****p ≤ 0.0001).

## Results

### Transcription activation requires a close spatial and orientational interplay between NP, PP2A-B56α, and VP30

**Figure 2.**
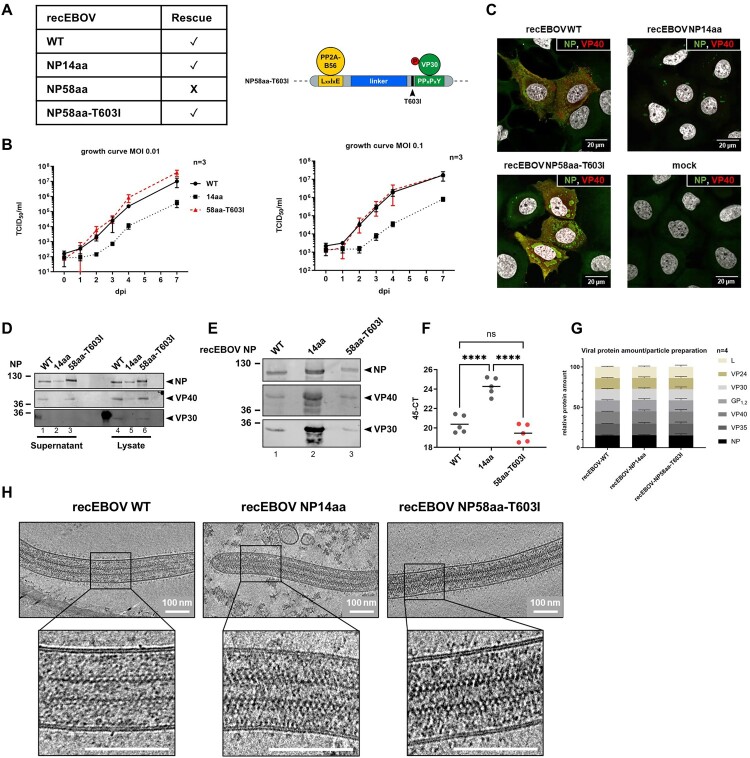
Rescue and characterization of recEBOV-NP mutants with an increased distance between the binding sites of PP2A-B56α and VP30. (A) Successfully rescued recEBOV as indicated. Bottom, scheme of NP with the naturally emerged compensatory mutation NP-T603I in the recEBOV-NP58aa context. (B) Growth kinetics of the in panel A introduced recEBOVs. VeroE6 cells were infected with the indicated MOI and supernatant was collected at 0-, 1-, 2-, 3-, 4-, and 7-dpi. Viral titres were determined by TCID_50_/ml and a mean from three independent experiments is shown. (C) Viral inclusion body formation in HuH7 cells infected with the respective viruses (MOI 0.3) at 24 hpi. NP and VP40 were stained with specific antibodies, and nuclei are stained with DAPI. (D) Protein expression in lysates and supernatants at 24 hpi from the samples generated in (C). (E) Viral protein incorporation in equal amounts of infectious virus particles (PFU). 2 × 10^5^ PFU were concentrated by ultracentrifugation for each of the respected recEBOVs and analysed by WB. (F) Viral genome copies in equal amounts of infectious virus particles (PFU). Mean of 5 individual experiments is shown. *C_t_* values (as 45–*C_t_*) are shown based on EBOV-specific RT-qPCR. (G) Ratio of incorporated viral proteins in recEBOV purified from HuH7 cells determined by MS/MS. Mean and standard deviation determined from four independent experiments. For individual measurements, see supplement S4. (H) Cryo-electron microscopy images of recEBOVs purified from HuH7 cells.

To investigate the role of EBOV NP as a connector that bridges the phosphatase PP2A-B56α with its substrate VP30, the spatial limitations within NP for efficient VP30 dephosphorylation and hence transcription activation were determined. The distance between PP2A-B56α and VP30 binding sites in NP (38aa) was increased by introducing a rigid linker (EAAAK)n. We constructed three NP mutants containing additionally either 14 (NP14aa), 24 (NP24aa), or 58 (NP58aa) amino acids (aa), respectively (Figure 1A). Lloviu virus (LLOV, genus *Cuevavirus*), the so far only member of the family *Filoviridae* found in Europe, was shown to have a reversed orientation of the two binding sites on NP [23, 33–36]. To determine orientation-related effects of PP2A-B56α and VP30 binding to NP, one EBOV NP mutant was constructed with exchanged binding sites (NPswitch, Figure 1A). Additionally, NP mutants with alanine mutations in their PP2A-B56α or VP30 binding motifs were included as negative controls (NPd.B56 and NPd.VP30) [23]. All NP mutants demonstrated typical NP-induced inclusion body formation as sites of viral RNA synthesis (Supplement S1A). The activity of our generated NP mutants was then assessed using an EBOV-specific transcription and replication competent virus-like particle (trVLP) assay (Figure 1B, Supplement S2). This assay is based on a tetracistronic EBOV-specific MG that in addition to the luciferase reporter expresses EBOV VP40, GP_1,2_, and VP24 [12, 30]. Luciferase activity in p0 cells revealed slightly improved EBOV-specific transcription for all NP mutants with increased B56α-VP30 distance independent from the length (NP14aa, NP24aa, and NP58aa) (Figure 1B). Viral replication was not affected, as the same improvement was observed when comparing the NP mutants using a monocistronic MG and a replication-deficient MG in parallel (Supplement S1B). These results confirmed that MG encapsidation and viral RNA synthesis were not affected by the introduced aa in NP. The same was true for the NPswitch mutant with the interchanged orientation of the PP2A-B56α and VP30 binding motifs. Also, NPd.30 supported reporter gene activity (Figure 1B) confirming previous results [37]. In contrast, transcription activity was abrogated in the case of NPd.B56 since VP30 remains hyperphosphorylated and hence transcriptionally inactive [23]. Infection of naïve p1 cells with trVLPs containing the NP mutants showed a drastic reduction in reporter gene activity compared to NPwt. This defect in supporting primary viral transcription was correlated with increased distance between PP2A-B56α and VP30 binding sites on NP (Figure 2C). Similarly, the NPswitch mutant was incapable to support primary viral transcription as well as both interaction-deficient NPd.B56 and NPd.VP30 mutants. Comparable expression of viral proteins in p0 cells and incorporation into trVLPs were verified by WB (Figure 1D, lanes 1–5). As expected, expression of VP40 in p0 cells is not detectable through WB due to expression from the tetracistronic MG that is driven by the viral polymerase complex in contrast to the viral proteins NP, VP30, VP35, and L that are coded on eukaryotic expression plasmids. Interaction of the NP mutants with either PP2A-B56 or VP30 was controlled in co-immunoprecipitation analyses. Quantification revealed that the interaction of NP mutants with PP2A-B56α (Figure 1E, lanes 10–12) and VP30 (Figure 1F, lanes 9–11) was not significantly affected upon increased B56α-VP30 distance (NP14aa to NP58aa), albeit the assay is prone to high standard deviations. In contrast, NPswitch was deficient in both PP2A-B56α and VP30 binding (Figure 1E, lane 9; Figure 1F, lane 12) suggesting that the orientation as well as neighbouring residues of both binding motifs are essential for the interaction within the trimeric complex. Finally, we assessed the impact of NP mutations on the efficiency of VP30 dephosphorylation mediated by an interaction with NP [19]. VP30 was expressed together with the respective NP mutants, and phosphorylation of VP30 was detected using a previously described VP30 phospho-specific antibody [20, 23]. Quantification of phosphorylated VP30 in relation to total amounts of VP30 revealed enhanced VP30 phosphorylation in the absence of NP (Figure 1G, lane 1), while VP30 phosphorylation is strongly reduced in the presence of NPwt due to the recruitment of endogenous PP2A-B56α towards VP30 (Figure 1G, lane 2) [20, 23]. Increasing the distance between the PP2A-B56α and VP30 binding sites in the different NP linker mutants led to a slight increase of VP30 phosphorylation (Figure 1G, lanes 4–6), in a length-dependent manner which likely accounts for the deteriorated primary transcription in p1 cells (Figure 1D). However, the increased phosphorylation was not as strong as in the absence of NP or compared to NP mutants lacking interaction to PP2A or VP30 (NPd.B56α or NPd.VP30 [23]), or both (NPswitch).

Together, these results suggest that the activation of primary transcription requires a close spatial and orientational interplay between PP2A-B56α and VP30 at the NP interface to allow efficient VP30 dephosphorylation.

### Compensatory mutation NP-T603I fully restores attenuation of recEBOV-NP58aa

The spatial-dependent interplay between NP, PP2A-B56α and VP30 was assessed next in an authentic viral life cycle through the generation of recombinant (rec)EBOV with NP mutations NP14aa and NP58aa. While we succeeded in rescuing recEBOV-NP14aa having introduced the shortest distance, rescuing a recEBOV-NP58aa with the longest distance between the PP2A-B56 and VP30 binding site was not possible (Figure 2A), which could be expected from our reporter gene assays (Figure 1C). However, in one single experiment, eventually cytopathic effect was developed in the case of recEBOV-NP58aa. Sequencing revealed the emergence of only one single point mutation within the whole recEBOV-NP58aa genome (corresponding to nt 2277 in recEBOV-NPwt). Intriguingly, this single point mutation was located in the NP gene, namely between the PP2A-B56 and VP30 binding sites where the 58aa linker was introduced (Figure 2A, right). The emergence of this single compensatory mutation changes NP threonine 603 (position in NPwt) into isoleucine (T603I), in direct neighbourhood to the VP30-PPxPxY motif (606–611). We hypothesized that this single compensatory mutation successfully rescued recEBOV-NP58aa-T603I. Growth kinetics in comparison to recEBOV-NPwt demonstrated attenuated replication of recEBOV-NP14aa, independently of the initial multiplicity of infection (MOI) (Figure 2B). Interestingly, while the NP58aa linker insertion severely attenuated primary transcription and was lethal for recEBOV-NP58aa generation, recEBOV-NP58aa-T603I showed no attenuation and reached viral titres similar to recEBOV-NPwt from day 1 post-infection (Figure 2B). The sequence of the outcoming recEBOVs at day 7 post infection was verified to exclude further compensatory mutations within the NP gene during propagation.

We then characterized the recEBOVs with respect to differences in morphology and particle composition that might contribute to the attenuation of recEBOV-NP14aa and to the recovery of recEBOV-NP58aa-T603I. Basic inclusion body formation, a hallmark during EBOV infection, was not affected in single plasmid-driven overexpression of NP58aa-T603I (Supplement S3). During recEBOV infection, large pleomorphic inclusions and VP40 in peripheral clusters and filopodia – typical for late stages of infection – were detected for recEBOV-NPwt and recEBOV-NP58aa-T603I (Figure 2C). In contrast, infection with recEBOV-NP14aa demonstrated smaller, punctuated inclusion bodies and VP40 localization at the plasma membrane was barely detectable, which suggests a very early state of infection. This was confirmed by WB, showing clearly reduced recEBOV-NP14aa in cell lysates and supernatants (Figure 2D lane 2, 5). VP30 could not be detected in the supernatants of the infected cells, independently of the investigated recEBOV, likely due to the overall low quantity of viral particles. We also analysed viral protein and viral RNA in equal amounts of infectious viral particles (determined by plaque-forming units, PFU). While protein amounts of NP, VP40, and VP30 and viral genome copies were comparable for recEBOV-NPwt and recEBOV-NP58aa-T603I (Figure 2E, lanes 1 and 3, Figure 2F), the amount of viral proteins and RNA was increased in recEBOV-NP14aa (Figure 2E, lane 2, Figure 2F). To investigate whether the ratio of incorporated viral proteins into the respective recEBOV was altered, purified virions were subjected to mass spectrometry. Comparing the mean fold change of peptides for the seven structural EBOV proteins across recEBOV, mutants showed no differences (Figure 2G, Supplement S4), indicating that the introduced mutations did not affect individual viral protein incorporation. These data suggest an increased quantity of defective infectious particles in the case of recEBOV-NP14aa. Finally, cryo-electron tomography of purified recEBOVs was performed to investigate their particle morphogenesis. All three recEBOVs demonstrated the typical filamentous shape of comparable length with regular GP_1,2_ decoration on their surfaces and showed similar condensed and decorated viral nucleocapsids, with no noticeable differences in particle morphology despite the introduced NP mutations (Figure 2H).

Together, these results show that EBOV amplification correlates with the spatial proximity of PP2A-B56α and VP30 binding to NP. Separating the PP2A-B56α and VP30 binding sites in NP resulted in an attenuated virus and overall reduced infectious virus particles compared to recEBOV-NPwt. Additionally, our data indicated that the emergence of the compensatory mutation in NP58aa-T603I during the rescue fully restored the viral growth of an otherwise non-viable recEBOV-NP58aa towards recEBOVwt.

### Characterization of the compensatory mutation NP-T603I in life cycle modelling systems

**Figure 3.**
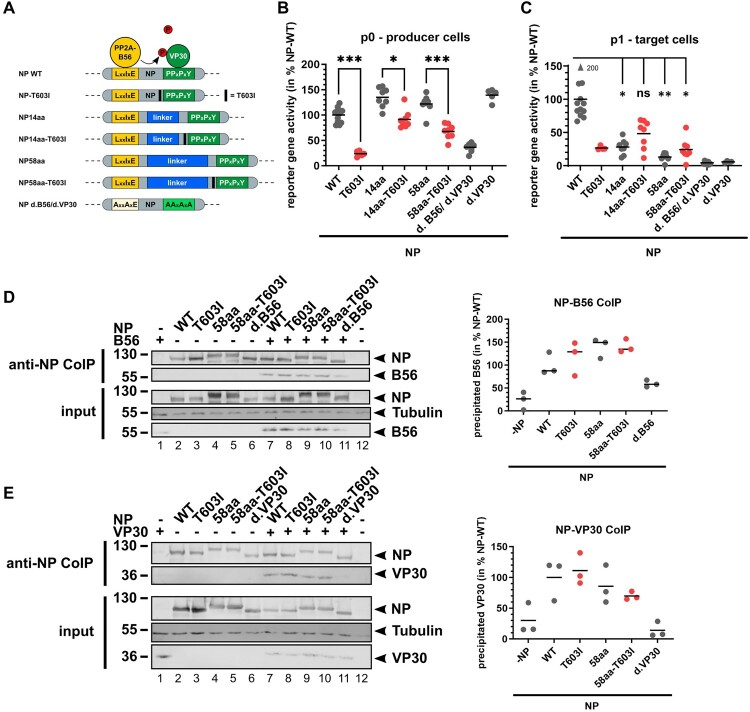
Impact of NP-T603I in life cycle modelling assays. (A) Scheme of analysed NP mutants. (B) trVLP assay with a tetracistronic minigenome, reporter gene activity in p0 cells. 72 hpt, luciferase activity was measured reflecting EBOV-specific transcription. NPwt set to 100%. Mean of individual experiments, *: *p* ≤ 0.05, ***: *p* ≤ 0.001 (C) Reporter gene activity in infected target cells (p1). Generated trVLPs from (B). Supernatants were collected 72 hpt, and trVLPs were purified by ultracentrifugation and used to infect naïve target (p1) cells. Luciferase activity reflects primary transcription. NPwt set to 100%. Mean of individual experiments. *: *p* ≤ 0.05, **: *p* ≤ 0.01. (D–E) CoIP of NP mutants with B56α (D) or VP30 (E) in HEK293 cells using epitope tagged magnetic beads for NP. On the right side, quantification of bands from individual experiments. NPwt set to 100%.

**Figure 4.**
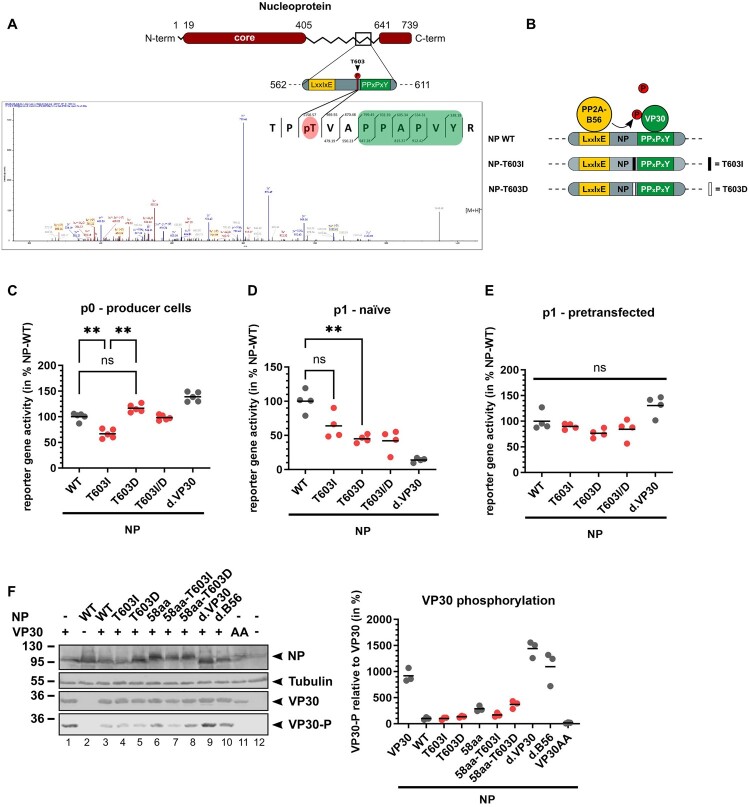
Impact of NP-T603 phosphorylation. (A) Mass spectrum of the NP peptide precursor ion-containing phosphorylated NP-T603, which was identified in recEBOV-NPwt virions purified from Huh7 cells. The schematic drawing in the top right corner shows the mass spectrometrically identified N- and C-terminal fragment ions of the phosphorylated peptide, whereby the small numbers indicate the calculated m/z values of the corresponding fragments, which were experimentally observed. (B) Scheme of NP-T603 phosphomutants including the mutants NP-T603I and NP-T603D. (C) trVLP assay with a monocistronic minigenome, reporter gene activity in p0 cells. 72 hpt, luciferase activity was measured reflecting EBOV-specific transcription. NPwt set to 100%. Mean of individual experiments, **: *p* ≤ 0.01 (D) Reporter gene activity in naïve target cells (p1). Generated trVLPs from (B) supernatants were collected to infect p1 cells. Luciferase activity reflects primary transcription. NPwt set to 100%. Mean of individual experiments. **: *p* ≤ 0.01. (E) Reporter gene activity in infected target cells that were previously transfected with plasmids encoding NPwt, VP35, VP30, L, and Tim-1 prior to infection with trVLPs as described in (D). Reporter gene activity reflects secondary transcription. NPwt set to 100%. (F) VP30 phosphorylation in the presence of NP upon recombinant expression in HeLa cells. VP30AA was included as positive control for dephosphorylated VP30 (lane 10 [20]). On the right side, quantification from individual experiments. NPwt set to 100%.

The impact and function of the compensatory mutation NP-T603I were further analysed in life cycle modelling assays by additional introduction into our previously investigated NP mutants and NPwt, as indicated (Figure 3A). Interestingly, mutation of T603I in the NPwt context abrogated reporter gene activity in p0 cells (Figure 3B). As before, reporter gene activity was increased upon the introduction of additional aa in NP compared to NPwt (Figure 3B, grey data points). Intriguingly, additional mutation of T603I in those mutants (Figure 3B, red data points) had a negative impact on viral transcription and restored reporter gene activity back towards NPwt levels. After infection of naïve p1 cells, reporter gene activity remained low upon T603I introduction in NPwt (Figure 3C), since particle formation is directly influenced by MG-dependent VP40, GP_1,2_, and VP24 transcription in p0 cells. In contrast, when comparing NP linker mutants with the additional mutation T603I to their counterparts lacking the mutation, the additional mutation was beneficial for activating primary viral transcription in p1 cells even though not fully reaching NPwt levels (Figure 4C, red data points). Interaction of NPwt or NP58aa-T603I with PP2A-B56α or VP30 revealed no statistically relevant differences upon additional introduction of T603I in the NP mutants (Figure 3D, and E, lanes 7–11, quantification on the right side). The same was true for interaction with VP40 or VP24 (Supplement S6).

Together, these results suggested a compensatory effect of T603I mutation for primary transcription in infected target cells upon increased distance between PP2A-B56α and VP30 binding.

### Dynamic phosphorylation of NPwt-T603 is important for primary viral transcription

In order to elaborate further on the relevance for NP-T603I in the viral life cycle that could contribute to the compensatory effect, purified recEBOV-NPwt virions were subjected to LC-MS/MS and analysed for post-translational modifications. Interestingly, T603 was found to be phosphorylated among several other NP phosphorylation sites (S13, T396, S413, T601, S639), (Figure 4A, Supplement S5). To investigate the impact of NPwt-T603 phosphorylation, we generated phosphomimetic NPwt mutants to assess constant phosphorylation (T603D) or dephosphorylation (T603I) (Figure 4B). Viral transcription activity was measured in a trVLP assay based on a monocistronic luciferase-containing MG together with the individual plasmid-driven expression of VP40, GP_1,2_, and VP24 [31]. Here, overall trVLP production is not relying on efficient MG transcription in p0 cells. When NPwt was substituted with the phosphomimetic NP-T603I or NP-T603D mutants, reporter gene activity was again reduced in the case of NP-T603I (Figure 4C), while NP-T603D increased reporter gene activity even higher than NPwt. This indicated that phosphorylation of NP-T603I directly affects viral transcription. The combination of both phosphomimetic mutants (NP-T603I/D) balanced reporter gene activity towards NPwt levels suggesting different roles of dynamic phosphorylation during viral transcription. This became even more evident during primary transcription in infected p1 cells, where both phosphomimetic NP mutants as well as the combination of both were clearly attenuated (Figure 4D). To confirm that replication of minigenomes as well as intruding trVLPs and MG template were generally functional in the presence of NP mutants, p1 cells pretransfected with RNP proteins NPwt, VP35, VP30, and L as well as the attachment factor Tim-1 were infected in parallel. Using this setting, we could confirm that the intruding MG templates in the case of the NP-T603 phosphomimetic mutants were generally transcription and replication competent as reporter gene activities of all mutants could be rescued by the presence of wt RNP proteins (Figure 4E). This suggested that a dynamic phosphorylation of NP at position T603 is important for primary viral transcription.

We again used the VP30 phospho-specific antibody for the detection of phosphorylated VP30 in the presence of the different NP-T603 mutants [20]. NPwt with either T603I or T603D showed no significant effect on VP30 phosphorylation (Figure 4F). In contrast, VP30 phosphorylation was again increased in the presence of NP58aa when compared to NPwt. Introduction of phosphomimicking T603D or dephosphorylated T603I in this context conversely affected VP30 phosphorylation as it was increased in the presence of NP58aa-T603D and decreased in the case of NP58aa-T603I (Figure 4F, lanes 6–8). Interaction of these mutants with VP40 or VP24 was unaffected (Supplement S6). Together, this suggests that dephosphorylation of NP58aa at T603 promotes VP30 dephosphorylation, which might explain the beneficial effect of the T603I mutation and successful rescue in case of the otherwise lethal recEBOV-NP58aa.

## Discussion

Reversible protein phosphorylation presents a frequent post-translational modification that governs cellular signalling pathways and also plays an important role during viral infections [38–42]. Viruses like EBOV exploit host cell kinases and phosphatases to regulate their viral protein phosphorylation, thereby influencing their distinct functions. Primary viral transcription during the EBOV viral life cycle is relying on dynamic phosphorylation of VP30 mediated by the cellular proteins PP2A-B56α and SRPK1 [19, 20, 23, 28]. We could previously show that NP emerged as a third key player in the regulation of filoviral VP30 dephosphorylation by recruiting both, the host phosphatase PP2A-B56α and VP30 to the interface of EBOV as well as MARV NP in order to allow VP30 dephosphorylation [23, 24]. Interestingly, both binding sites reside in the C-terminal IDR of NP and are separated in EBOV NP by only 38 aa. We could show that increasing the distance between both binding sites at the EBOV NP interface correlated, as expected, with increased VP30 phosphorylation, and consequently inefficient primary transcription. This was also evident by a clearly attenuated recEBOV-NP14aa. This attenuation of recEBOV-NP14aa is likely a result of insufficient VP30 dephosphorylation and as a consequence reduced primary transcription upon target cell infection. Data from life cycle modelling experiments and the unaltered particle morphology as well as their protein composition further support this hypothesis. Intriguingly, a compensatory point mutation in NP at position T603 emerged during the rescue of an otherwise non-viable recEBOV-NP58aa. Sequencing revealed the occurrence of the mutation in passage 3, while additional attempts to cultivate recEBOV from passage 1 or 2 of the same experiment remained unsuccessful. Strikingly, the compensatory mutation T603I fully restored viral transcription, replication and particle release of recEBOV-NP58aa to recEBOV-NPwt levels. The additional T603I mutation in the NP linker mutants was also beneficial for primary viral transcription in infected target cells using life cycle modelling assays, albeit it did not fully restore primary transcription to the NPwt level. Binding affinities of NP mutants to either VP30 or PP2A-B56α were not affected upon additional T603I mutation, but VP30 dephosphorylation was improved in the presence of T603I compared to phosphomimicking T603D or wildtype T603. These findings indicate that the compensatory mutation T603I in recEBOV-NP58aa evolved to restore efficient VP30 dephosphorylation, proposing that (de)phosphorylation of NP affects (de)phosphorylation of VP30. Reversible phosphorylation of VP30 is absolutely essential for virus rescue and amplification [19]. Here, the introduction of alanine mutations at the EBOV VP30 phosphosites was lethal for the generation of a recEBOV, while a single reintroduction of a dynamically phosphorylatable serine 29 restored viral replication to recEBOVwt without attenuation, like recEBOV-NP58aa-T603I. This suggests that the regulated balance of VP30 reversible phosphorylation is critical for an efficient viral life cycle and can determine failure or success with respect to virus rescue. The emergence of the compensatory mutation further emphasizes a strong evolutionary pressure on the intact interplay between NP, PP2A-B56α, and VP30 and subsequent VP30 dynamic phosphorylation.

Interestingly, NP-T603 was phosphorylated in purified recEBOV from infected HuH7 cells. Upon mutational analysis towards phosphomimetic NPwt-T603 mutants, basic reporter gene activity was impaired upon a nonphosphorylated T603I, while it was unaffected upon phosphomimetic T603D. The presence of both phosphomimetic versions mitigated the negative effect suggesting distinct functions of dynamic T603 phosphorylation during transcription. However, upon infection of naïve target cells, dynamic phosphorylation of T603 at the same NP molecule was important, as none of the phosphomimetic NP mutants was able to establish primary viral transcription like NPwt. VP30 phosphorylation was not affected upon phosphomutations at NPwt-T603 suggesting an VP30-independent, so far unknown function of NP-T603 phosphorylation during the viral replication cycle. Several threonine and serine residues were already identified as phosphosites within EBOV NP, with the majority residing in its IDR [43]. However, the functional role of phosphorylation as post-translational modification of EBOV NP was so far not studied. In our study, we could confirm two phosphosites (Supplement S5, marked with an asterisk) and expand the list of potentially phosphorylated residues by four further sites (no asterisk). Among the phosphosites, also T603 was phosphorylated in EBOV particles purified from VeroE6, but not from bat-derived EpoNi/22.1 cells. Another study identified phosphorylation of stably-expressed NP in HEK293 cells, which could not detect phosphorylation of T603 [44]. These data suggest on the one hand that phosphorylation sites in NP may be cell type-dependent. However, it is also feasible that NP phosphosites differ between cell-associated and virion-associated NP, which implicates a regulatory function of dynamic NP phosphorylation during nucleocapsid assembly.

Also, other negative-strand RNA viruses exploit host phosphatases to modulate viral protein functions. During RSV infection, dephosphorylation and hence activation of the transcriptional antiterminator M2-1 is accomplished by a regulated interplay with host phosphatase PP1. Interestingly, RSV-P serves, like EBOV NP, as a scaffold that bridges the interaction between phosphatase and its substrate M2-1. RSV-P harbours several phosphosites required for viral RNA synthesis and interactions with viral proteins [42, 45, 46]. In particular, phosphorylation of P T108 prevented binding to M2-1, which subsequently impaired viral transcription due to inefficient M2-1 dephosphorylation at the P interface [47], similar to our results. Phosphorylation of P-S54 negatively affects the interaction with the matrix protein M. This phosphorylation is suggested to liberate viral RNPs from M protein during the uncoating of the intruding virions. PP2A is involved in its control, while the cellular kinase is unknown [48]. Whether EBOV NP-T603 phosphorylation fulfils similar regulatory functions during uncoating like RSV-P, or whether phosphorylation of NP influences its oligomerization and RNA binding remains to be determined in future studies [38, 49–52].

Cellular phosphatase PP1, previously shown to enable VP30 dephosphorylation in addition to PP2A [15, 18, 23], was shown to interact with EBOV NP. It is suggested that the interaction of NP with PP1 is important for NP dimerization and EBOV capsid formation. However, whether PP1 also contributes to NP dephosphorylation, thereby modulating its functions is so far unknown. Interestingly, during PP1 inhibitor treatment in EBOV-infected cells, the compensatory mutation NP-E619 K emerged that reinforced the binding of PP1 to NP [53]. Again, this compensatory mutation was located next to the VP30 binding site in the NP IDR [23, 26, 28, 37, 54] emphasizing the evolutionary pressure in this region to ensure efficient viral replication. Additionally, the NP IDR itself might have evolved to provide a dynamic and flexible platform for molecular interactions [55]. The inherent flexibility of IDRs would allow EBOV NP to adapt to various binding partners, enabling a diverse range of biological functions essential during the viral life cycle [55].

**Figure 5.**
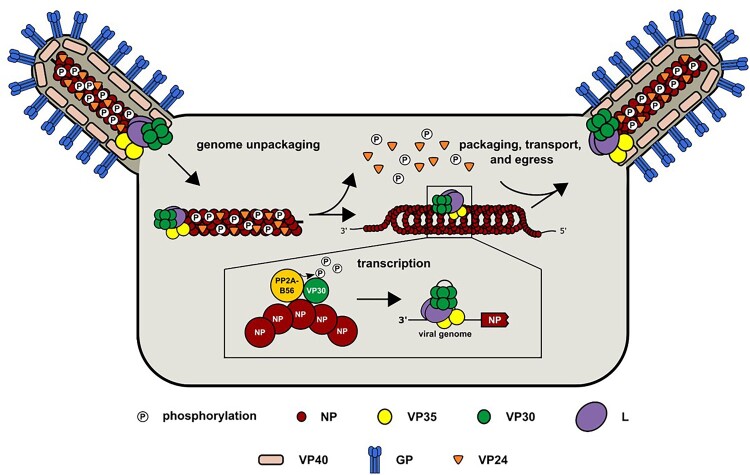
Model of NP and VP30 phosphorylation contributing to an efficient EBOV life cycle. NP phosphorylation state differs between virions and in cells. EBOV NP is phosphorylated at position T603 in virions, upon infection of target cells; NP-T603 requires dephosphorylation to support efficient viral transcription and replication. At a later time point during the viral replication cycle, NP-T603 is re-phosphorylated and finally packaged into newly formed virions. NP serves as a key player in the tight interplay of VP30-dependent transcription initiation by facilitating VP30 dephosphosphorylation through PP2A-B56α.

In summary, our data emphasize the essential role of NP in the complex interplay involved in VP30-dependent transcription initiation (Figure 5). Our findings also suggest that dynamic phosphorylation of NP at T603, which differs between virion and cells, is critical for primary viral transcription. Further investigation is required to determine if NP phosphorylation also impacts (un)packaging, transport or egress of nucleocapsids. The emergence of the compensatory mutation NP-T603I highlights the flexibility of the NP C-terminal IDR, which is essential for NP’s diverse interaction with viral and host proteins. This flexibility significantly contributes to NP’s multifaceted roles at different stages of EBOV infection.

## Supplementary Material

Supplemental Figures_revised.pdf

Supplemental Methods_revised.docx
