# Supplementary material for: To be or not to be phosphorylated: understanding the role of Ebola virus nucleoprotein in the dynamic interplay with the transcriptional activator VP30 and the host phosphatase PP2A-B56
Source: Emerg Microbes Infect. 2024 Dec 27;14(1):2447612. doi: 10.1080/22221751.2024.2447612 (PMC11727051; doi:10.1080/22221751.2024.2447612)
Supplement: Supplemental Figures_revised.pdf [file TEMI_A_2447612_SM4687.pdf]

Supplement S1

A

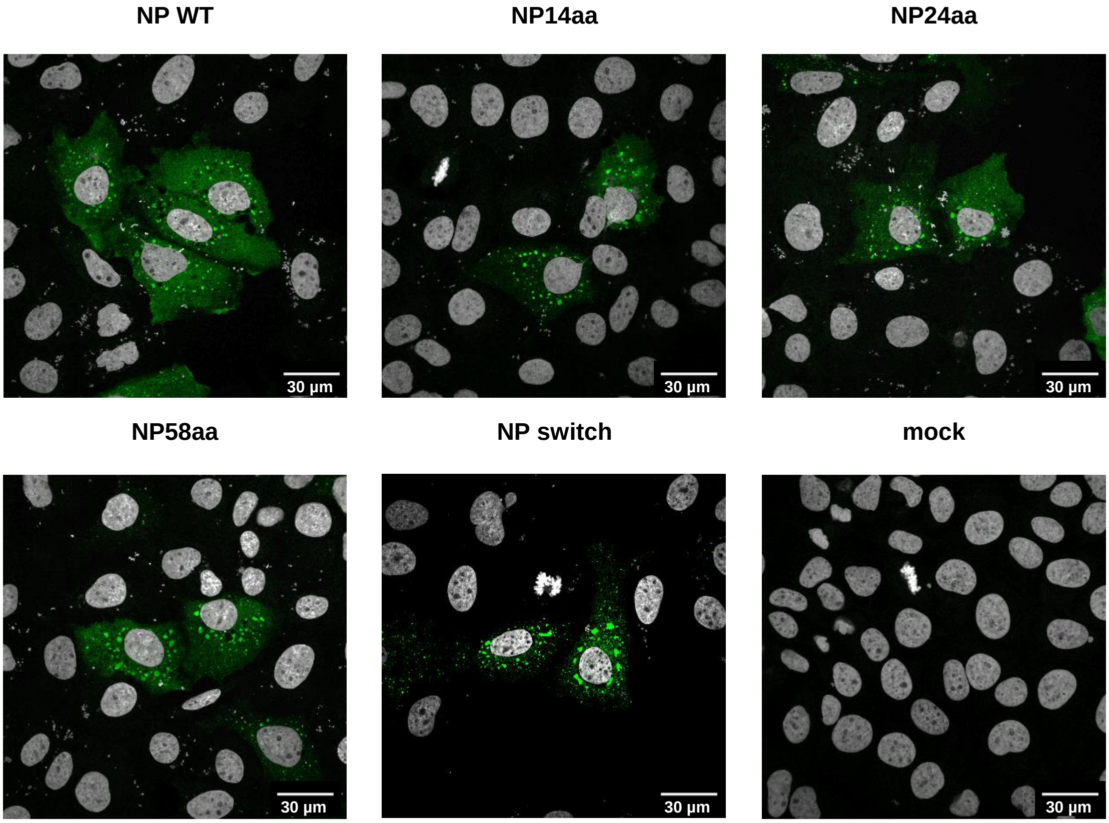

B

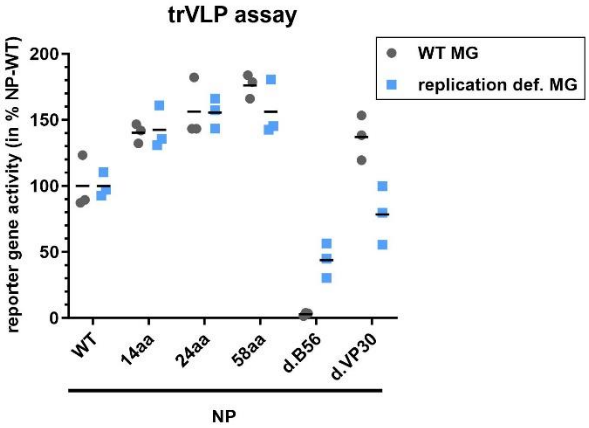

S1 (A) Inclusion body formation of the NP mutants introduced in Fig 1A. HuH7 cells were transfected with expression plasmids encoding for the respective NP mutants. Cells were fixed 24 hpt and stained against EBOV NP (α-NP (chicken) and α-chicken Alexa 488) (green). Cell nuclei were stained with DAPI (grey). Pictures were taken with a Stellaris 8 confocal microscope and processed with ImageJ. The immunofluorescence was performed in three independent experiments and a representative picture of bar = 30 µM. **(B)** EBOV-specific trVLP assay performed in HEK293 cells with the different NP mutants. Either the WT MG or a replication-deficient MG was used (deletion of 55 nts from the trailer [10]). Cells were lysed 72 hpt, reporter gene activity was measured, normalized to the firefly luciferase expression control, and the mean of NP-WT was set to 100%. For statistical analysis, a one-way ANOVA with multiple comparisons was performed using GraphPad Prism.

Supplement S2

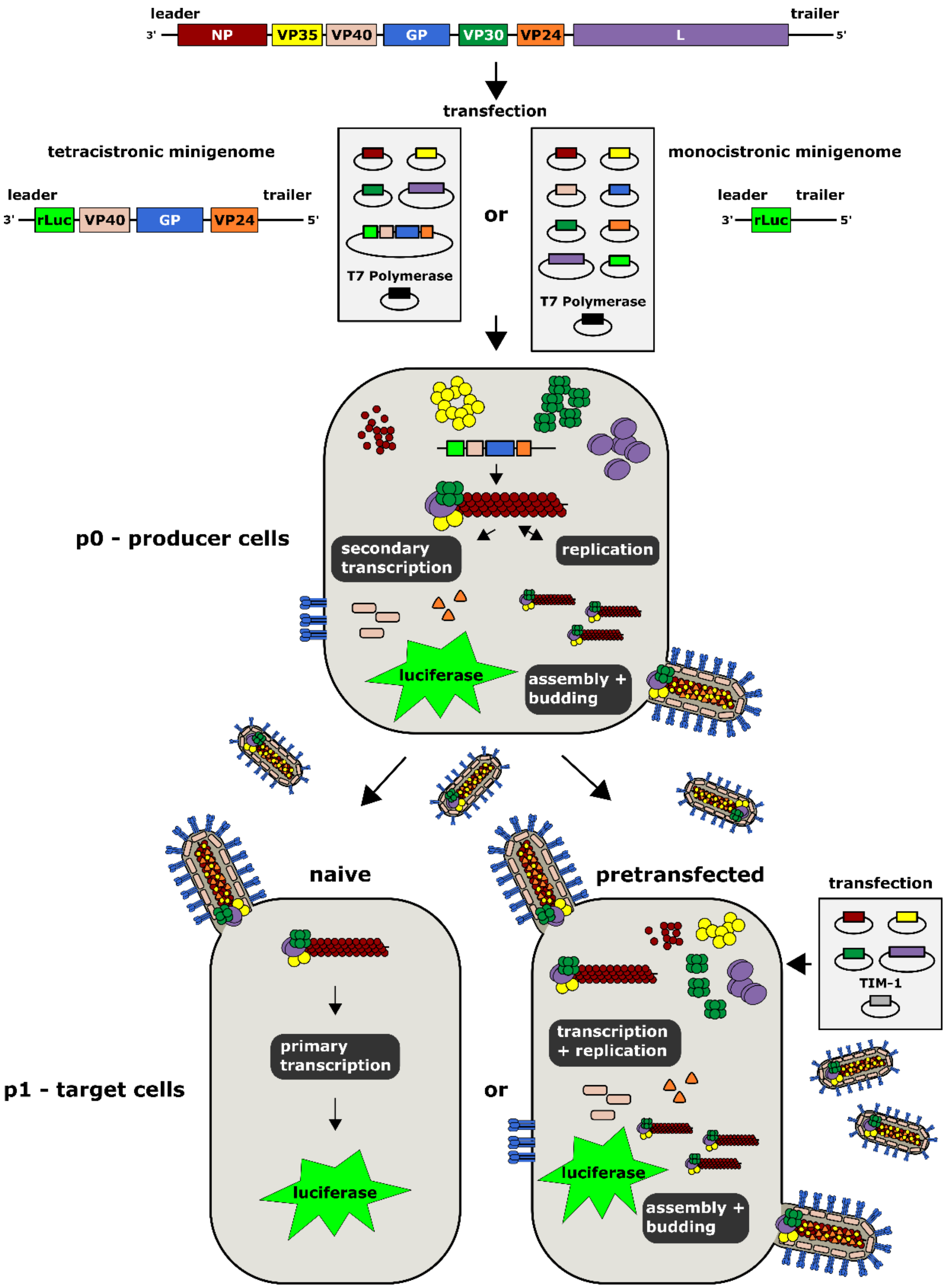

S2 Schematic of a trVLP assay. Instead of the full-length EBOV genome, a minigenome is utilized that codes for a luciferase reporter gene. Either a tetracistronic minigenome, additionally encoding VP40, GP, and VP24, or a monocistronic minigenome can be used. p0 cells are transfected with the respective minigenome and expression plasmids of the additional viral proteins. The viral RNPs recognize the transcribed minigenome leading to its transcription and replication. Viral particles are released from p0 cells, due to the expression of all viral proteins. These particles can be purified from the supernatant and used to infect either naïve p1 cells or pretransfected p1 cells that already express the RNPs.

Supplement S3

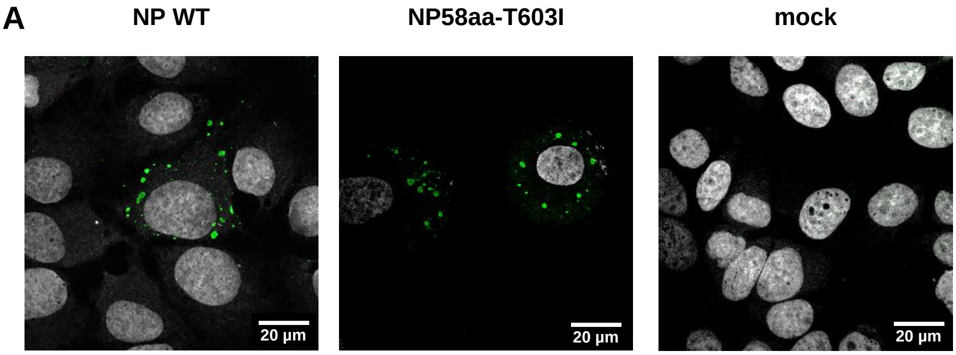

S3 (A) Inclusion body formation of the NP-58aa-T603I upon recombinant expression. HuH7 cells were transfected with pCAGGS expression plasmids encoding for the respective NP mutants. Cells were fixed 24 hpt and stained against EBOV NP (α-NP (chicken) and α-chicken Alexa 488) (green). Cell nuclei were stained with DAPI (grey). Pictures were taken with a Stellaris 8 confocal microscope and processed with ImageJ. The immunofluorescence was performed in three independent experiments and a representative picture of bar = 20 µM.

Supplement S4

Log2 intensities

Experiment_1

Uniprot ID	recEBOV	WT_rep1	WT_rep2	NP14aa_rep1	NP14aa_rep2	NP58aa-T603I-rep1	NP58aa-T603I-rep2
P18272	NP	28,84	28,71	25,02	24,82	22,18	22,53
Q05127	VP35	27,35	27,29	21,59	21,50	21,00	21,21
Q05128	VP40	28,68	28,81	23,30	22,77	21,74	21,97
Q05320	$GP_{1,2}$	25,33	25,49	20,57	20,87	19,52	19,61
Q05323	VP30	24,84	24,93	21,05	20,74	17,44	16,94
Q05322	VP24	25,66	25,68	20,67	19,67	21,38	21,39
Q05318	L	25,49	25,48	21,20	20,97	19,36	19,38

Experiment_2

Uniprot ID	recEBOV	WT_rep1	WT_rep2	NP14aa_rep1	NP14aa_rep2	NP58aa-T603I-rep1	NP58aa-T603I-rep2
P18272	NP	30,89	30,85	28,26	28,26	30,52	30,38
Q05127	VP35	29,29	29,33	26,28	26,35	29,20	29,27
Q05128	VP40	30,46	30,36	29,16	28,93	30,73	30,49
Q05320	$GP_{1,2}$	28,60	28,31	26,45	26,37	28,88	28,65
Q05323	VP30	28,40	28,36	25,30	25,24	28,11	27,97
Q05322	VP24	28,63	28,50	25,64	25,47	28,64	28,47
Q05318	L	29,40	29,34	25,75	25,53	28,17	28,04

Experiment_3

Uniprot ID	recEBOV	WT_rep1	WT_rep2	NP14aa_rep1	NP14aa_rep2	NP58aa-T603I-rep1	NP58aa-T603I-rep2
P18272	NP	29,37	29,39	26,31	26,33	29,48	29,54
Q05127	VP35	27,91	27,88	25,19	25,19	28,15	28,22
Q05128	VP40	29,94	29,89	26,83	26,78	29,82	29,89
Q05320	$GP_{1,2}$	25,52	25,59	23,75	23,75	26,47	26,69
Q05323	VP30	27,71	27,72	24,05	24,09	27,38	27,46
Q05322	VP24	26,82	26,79	23,27	23,33	27,26	27,30
Q05318	L	26,79	26,77	23,36	23,36	26,81	26,89

Experiment_4

Uniprot ID	recEBOV	WT_rep1	WT_rep2	NP14aa_rep1	NP14aa_rep2	NP58aa-T603I-rep1	NP58aa-T603I-rep2
P18272	NP	27,89	30,01	26,12	26,19	29,78	29,62
Q05127	VP35	26,27	29,42	25,43	25,35	29,22	29,18
Q05128	VP40	27,97	29,92	27,55	27,47	30,40	30,27
Q05320	$GP_{1,2}$	26,02	28,57	24,91	24,90	28,37	28,16
Q05323	VP30	25,76	28,09	24,12	23,98	28,24	28,24
Q05322	VP24	24,16	27,36	22,94	23,36	26,99	27,01
Q05318	L	24,96	28,80	23,62	23,76	27,68	27,53

S4 Log2 protein intensity values of structural EBOV proteins from rec.EBOV WT particles. Four independent biological experiments were measured by mass spectrometry in technical duplicates. Raw data from Figure 2G.

Supplement S5

Phosphorylated Amino acid in NP
S13
T396
S413
T601*
**T603***
S639

S5 Identification of phosphorylated NP residues in rec. EBOV WT particles via mass spectrometry**.** HuH7 cells were infected with rec. EBOV WT with an MOI of 0.2. Supernatant was collected 72 hours post-infection. Viral particles were purified from the supernatant by ultracentrifugation over a sucrose cushion, resuspended in PBS buffer and repelleted. Virus pellets were dissolved in 1% SDS and cooked. Protein identification in mass spectrometry was performed with a timsTOF Pro mass spectrometer (Bruker Daltonic) and the data was analyzed with Proteome Discoverer 2.4 and MaxQuant. 3 independent replicates (rep) with each 2 technical replicates were performed. Phosphorylation sites in NPwt were classified as valid if they were reliably detected by both analysis algorithms (Proteome Discoverer and MaxQuant) in at least two replicates (technical or biological). Residues marked with an (*) were already identified to be phosphorylated in viral particles in a study by Ivanov et al. from 2020.

Supplement S6

A

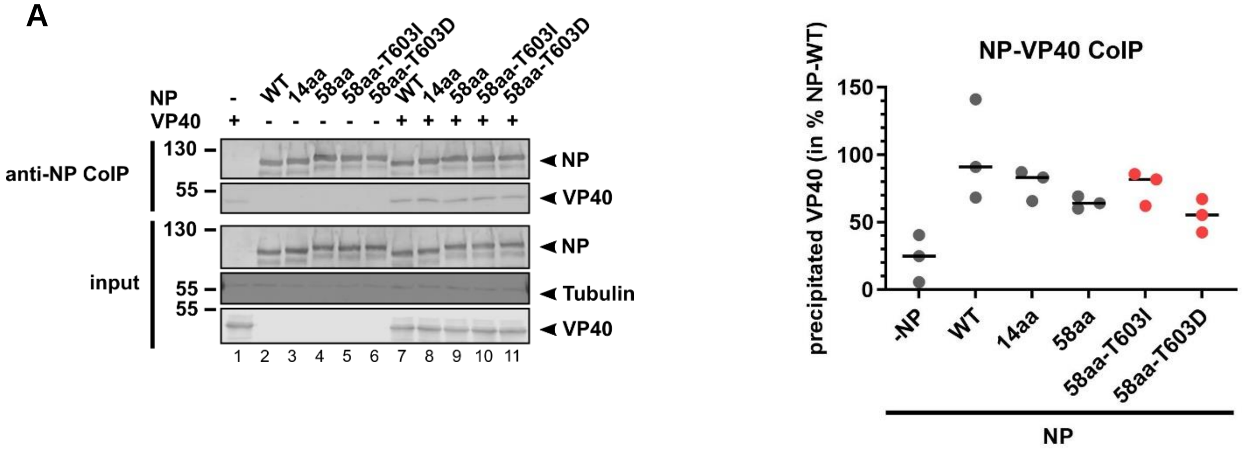

B

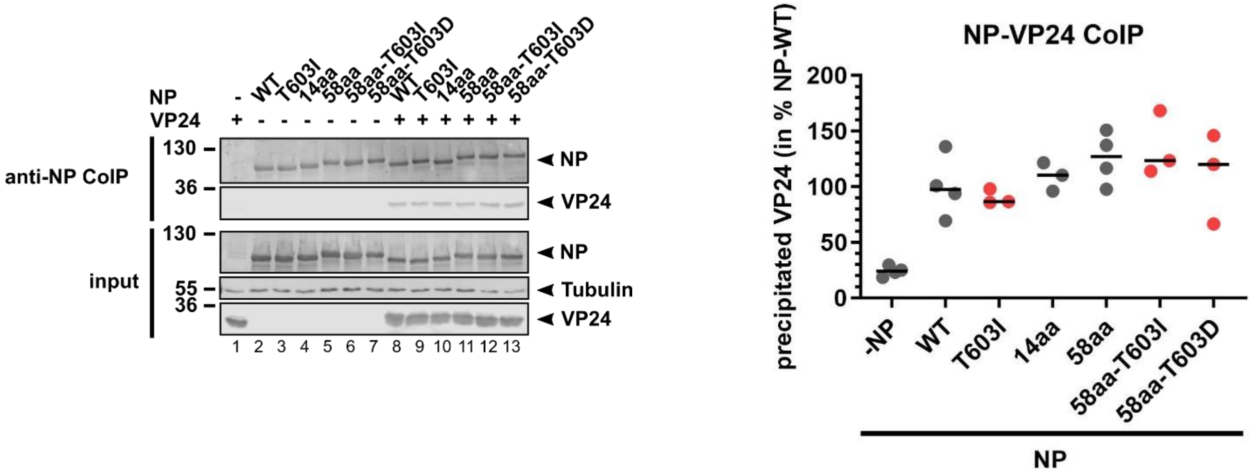

S6 Interaction of NP mutants with VP40 **(A)** or VP24 **(B)** in co-immunoprecipitation analysis. HEK293 cells were transfected with pCAGGS VP40 or VP24 expression plasmids, and the indicated flag-tagged NP mutants. Flag-tagged NP was precipitated 48 hpt with anti-flag antibody-covered magnetic beads, and subjected to WB analyses. Antibody staining with α-NP (chicken) and α-chicken LI-COR 780 nm; α-VP40 chicken and α-chicken LI-COR 780 nm; α-VP24 (rabbit) and α-rabbit LI-COR 780 nm, and α-tubulin (mouse) and α-mouse LI-COR 680 nm. Right: Quantification of VP40 or VP24 co-precipitated by NP. NP WT was set to 100%.
