# Supplementary material for: To be or not to be phosphorylated: understanding the role of Ebola virus nucleoprotein in the dynamic interplay with the transcriptional activator VP30 and the host phosphatase PP2A-B56
Source: Emerg Microbes Infect. 2024 Dec 27;14(1):2447612. doi: 10.1080/22221751.2024.2447612 (PMC11727051; doi:10.1080/22221751.2024.2447612)
Supplement: Supplemental Methods_revised.docx [file TEMI_A_2447612_SM4686.docx]

**Supplementary Methods**

**Co-Immunoprecipitation analysis**

8x10^5^ HEK293 cells per well were seeded in a 6-well plate 24 h prior to transfection. Flag-tagged NP or the indicated mutants were co-transfected with either VP30, B56-myc, VP24-HA, or VP40-HA from pCAGGS expression plasmids. 1 µg of each construct was transfected using 3 µl *Trans*IT^®^-LT1 (Mirus, 731-0029) per µg DNA. DNA amounts were adjusted with empty pCAGGS vector. CoIPs for B56a, VP40, and VP24 pull-down by NP were performed as follows. Cells were harvested and lysed 48 h post transfection for 20 min at room temperature in co-immunoprecipitation (CoIP) buffer (20 mM Tris/HCl pH 7.5, 100 mM NaCl, 1% (v/v) Nonidet P-40, 5 mM EDTA) supplemented with 0.1 % Triton X-100 and 1x c0mplete^TM^ (Roche). An aliquot of the cleared lysate was taken as expression control and the rest was incubated with mouse anti-flag M2 agarose (Sigma-Aldrich A2220) for 2 h rotating at 4°C. The bound proteins were washed 4x in CoIP buffer and finally resuspended in 45 µl 1x SDS sample buffer followed by SDS-PAGE and WB analysis. VP30-NP CoIPs were done using mouse anti-flag magnetic beads (MedChemExpress HY-K0207) according to the manufacturer’s recommendations. Cell lysates were incubated at room temperature for 10 min under constant rotation with the magnetic beads for protein binding, washed 4 times in TBS + 0.1% Tween and finally eluted with 45 µl 2x SDS sample buffer followed by SDS-PAGE and WB analysis.

**SDS-PAGE and Western Blotting (WB)**

Samples for SDS-PAGE and WB analysis were incubated for 10 min at 100°C in sodium dodecyl sulfate (SDS) sample buffer (100 mM Tris/HCl pH 6.8, 0.2% bromophenol blue, 20% glycerol, 10% 2-mercaptoethanol, 4% SDS). Afterwards, samples were separated by 10% SDS polyacrylamide gels and blotted on nitrocellulose membranes (Amersham, 0.45 µM, 10600002). Membranes were blocked in 10% skim milk in PBS_def_. Primary antibodies were diluted in 1% skim milk and incubated for 1 h at room temperature: rabbit anti-VP30 (1:500) [1], rabbit anti-VP30 P serine 29 (1:100) [2], mouse anti-tubulin (1:500, Sigma T9026), chicken anti-VP40 (1:500), chicken anti-NP (1:1000) [3,4], rabbit anti-VP24 (1:500, kindly provided by Viktor Volchkov), rabbit anti-myc (1:250, Cell Signaling 2272S), mouse anti-flag biotin M2 (1:1000, Sigma-Aldrich F9291), mouse anti-flag (1:1000, Sigma F1804). Secondary antibodies were diluted 1:15,000 in 1% skim milk and used accordingly; anti-chicken (LI-COR 926-32218, 780nm), anti-rabbit (Rockland 611-132-002, 780nm), anti-rabbit (LI-COR 926-68071, 680nm), anti-mouse (LI-COR 926-68072, 680nm). Blots were imaged and quantified using Odyssey CLx by LI-COR.

**EBOV-specific transcription and replication-competent virus-like particle (trVLP) assay**

The trVLP assay was performed as previously described [5,6]. Briefly, for tetracistronic trVLP assays 8x10^5^ HEK293 producer (p0) cells were transfected in a 6-well plate as described above for the CoIP analysis. pCAGGS plasmids coding for the viral ribonucleoproteins NP (125 ng), VP35 (125 ng), VP30 (75 ng), and L (1 µg) were transfected together with a T7-polymerase (250 ng), a fire fly luciferase for normalization (25 ng), as well as a tetracistronic minigenome (250 ng) that additionally to the nano luciferase reporter gene codes for the viral proteins VP40, GP_1,2,_ and VP24. In case of monocistronic trVLP assays pCAGGS plasmids coding for the viral proteins VP40 (250 ng), GP_1,2_ (250 ng), and VP24 (60 ng) were additionally transfected into p0 cells. A previously described monocistronic minigenome lacking 55 nts from the trailer was used as a replication deficient minigenome mutant [7]. p0 producer cells were harvested and lysed 72 h post transfection. Supernatant was collected and trVLPs were purified via ultra-centrifugation in an SW60 rotor over a 20% sucrose cushion for 2h at 40,000 rpm. Pelleted trVLPs were resuspended in PBS and either used to infect naïve HEK293 p1 indicator cells or HEK293 p1 cells that were pretransfected with pCAGGS plasmids coding for the viral proteins NP (125 ng), VP35 (125 ng), VP30 (75 ng), and L (1 µg) as well as the attachment factor Tim-1 (250 ng). p1 indicator cells were harvested and lysed 72 h post infection followed by renilla luciferase measurement. Results of NP WT were set to 100%. In order to detect protein incorporation into trVLPs, six wells of a 6-well plate were transfected for each sample. Supernatant of the 6-wells was combined and purified via ultra-centrifugation in two buckets of an SW41 rotor over a 20% sucrose cushion for 2h at 40,000 rpm. trVLP pellets were combined by resuspending them in PBS, an appropriate amount of 4x SDS sample buffer was added, and the samples were cooked followed by SDS-PAGE and WB analysis.

**Rescue of recombinant Ebola virus**

All experiments with recombinant (rec) EBOV were conducted under BSL4 conditions at the Philipps-University Marburg according to national regulations. A full-length plasmid (pAMP rgEBOV) was used as WT based on the EBOV Mayinga sequence (GenBank accession number AF086833). Rescues were performed on 2x10^5^ HuH7 cells per well seeded in a 6-well plate 24h prior to transfection. Transfections were carried out as described above (see EBOV-specific transcription and replication-competent virus-like particle assay) in duplicates including plasmids encoding for the viral proteins NP, VP35, VP30, and L, the full-length cDNA wt or NP14aa, NP24aa, or NP58aa (1 µg) and a pCAGGS T7 plasmid to enable initial vRNA synthesis. Transfection media was exchanged 24h post transfection followed by a blind passage on VeroE6 cells on day 7 post transfection. Cells were further monitored for the development of a cytopathic effect (CPE) in which case the rescue was further passaged on fresh VeroE6 cells in a T75 flask. For stock production, 20 ml of VeroE6 supernatant was cleared from floating cells by centrifugation for 10 min at 1700 rpm and 4°C followed by aliquotation. The stock virus was titrated by plaque assay on VeroE6 cells and calculated as PFU/ml. Full genome sequencing of the rescued recEBOV stocks was performed on an Illumina iSeq 100 system [8].

**Growth kinetics of recombinant Ebola virus**

8x10^6^ VeroE6 cells were seeded in T75 flasks and infected with a multiplicity of infection (MOI) of 0.1 or 0.01. Cell culture media was removed, and the appropriate amount of virus-containing supernatant was added in a total volume of 5 ml DMEM without FCS. The inoculum was incubated for 1 h at 37°C and 5% CO_2_ under periodic shaking. Subsequently, the inoculum was removed and exchanged with 20 ml of DMEM substituted with 3% FCS and Q+P/S. The infected cells were incubated again at 37°C and 5% CO_2_ and 1 ml of supernatant was taken at 0, 1, 2, 3, 4, and 7 days post infection. On day 7 post infection, RNA samples were additionally taken for RT-PCR and Sanger sequencing to confirm the introduced mutations within NP. Samples from the different time points were titrated in quadruplicates by TCID_50_ analysis in VeroE6 cells with 1:5 dilution steps as described previously [9]. Titers were calculated as TCID_50_/ml using the Spearman-Kärber method [10].

**Detection of phosphorylated VP30 by western blot analysis**

Phosphorylation state of VP30 was measured in HeLa cells [11]. HeLa cells were seeded at a density of 4x10^5^ cells per well of a 6-well plate 24 h prior to transfection with pCAGGS plasmids of flag-tagged VP30 WT and flag-tagged NP or the indicated NP mutants. 1 µg of each construct was transfected using 3 µl *Trans*IT®-LT1 (Mirus, 731-0029) per µg DNA. DNA amounts were adjusted with empty pCAGGS vector if necessary. Cells were harvested in 1 ml Tris-buffered saline (TBS) 48 h post transfection and pelleted by centrifugation (900xg, 2:30 min). The resulting pellet was lysed for 20 min at room temperature in 150 µl cell lysis buffer (Cell Signaling, diluted in TBS) substituted with 1x c0mplete^TM^ (Roche), 1:100 PMSF (Sigma-Aldrich), 1:100 Calyculin A 10µM (Merck/ Sigma-Aldrich) and 1:1000 MG-132 (Sigma-Aldrich). The lysate was cleared by centrifugation for 5 min at 17,000xg and the supernatant was mixed with 4x SDS sample buffer. Detection of phosphorylated VP30 at position 29 by a phospho-specific antibody (anti pS29 VP30, rabbit) was performed after SDS-PAGE and WB analysis as described in [2]. Complete VP30 was detected via a C-terminal flag-tag with a mouse anti-flag antibody (Sigma F1804). Blots were imaged using an Odyssey CLx by LI-COR and quantifications were performed with Li-Cor Odyssey Image Studio 5.2 followed by a one-way anova with multiple comparisons performed with Prism 10.0.2 (GraphPad) for statistical analysis.

**RT-qPCR of Ebola virus vRNA**

RT-qPCRs of viral RNA from recEBOVs were performed as previously described [12]. Briefly, cell culture supernatant was inactivated with AVL and 100% ethanol and extracted from the BSL4 facility. RNA was purified using the QIAmp Viral RNA mini kit from Qiagen following manufacturer’s instructions (ref. 52906). Following RT-PCRs were done using the RevertAid reverse transcriptase kit from Thermo scientific (ref. EP0442, Primer: CAAACCAGGTGTGATTACAGTAACAATT). Finally, the qPCR was performed using the Luna Universal qPCR Master Mix from NEB (ref. M3003L) following manufacturer’s instructions (Primer (5` to 3`): CAAACCAGGTGTGATTACAGTAACAATT and GCCGGACACACAAAAAGAAAGAA).

**Cryo-electron tomography acquisition and tomogram reconstruction**

5x10^6^ HuH7 cells were seeded in a T175 cell culture flask 24h pre infection. Cells were infected with an MOI of 0.2 for recEBOV WT and recEBOV NP58aa T603I and incubated three days prior to the purification of viral particles. To generate comparable amounts of virus particles from the attenuated recEBOV NP14aa, we infected cells with an MOI of 0.045 due to restrictions through a low stock titer and cultured them for 7 days before purification of viral particles. Supernatants were harvested and cleared of cell debris by centrifugation for 10 min at 1700 rpm. Viral particles in the cleared supernatant were subsequently pelleted by ultracentrifugation over a 20% sucrose cushion in HNE buffer (10 mM HEPES, 100 mM NaCl, 1 mM EDTA, pH 7.4) in a SW32 rotor for 1:30 h at 25,000 rpm. The virus pellet was resuspended in HNE buffer and once more pellet for 20 min at 14,000 rpm. Finally, the pellet was twice flooded with fixation buffer (4% PFA, 0.1% glutaraldehyde, in HNE buffer) and extracted from the BSL4 facility [13].

For cryo-electron tomography chemically fixed EBOV preparations were 1:10 diluted and mixed with 10 nm protein A‐coated colloidal gold (Aurion). 3 µL of the sample were applied onto glow‐discharged EM grids (200 mesh, R 2/1, Quantifoil) and vitrified using a GP2 plunge freezer (Leica) at an ethane temperature of −183°C and 95% chamber humidity. Tilt series were collected on a Titan Krios Transmission Electron Microscope (ThermoFisher Scientific) operated at 300 keV and equipped with a BioQuantum® LS energy filter with a slit width of 20 eV and K3 direct electron detector (Gatan) using SerialEM [14]. Tilt series were acquired at 33,000 magnification (pixel size 2.671 Å) using a dose‐symmetric acquisition scheme [15] with an electron dose of approximately 3 e−/Å2 per projection with tilt ranges from 60° to −60° in 3° increments using SerialEM [14] and a scripted dose‐symmetric tilt‐scheme [15]. Tomograms were reconstructed using the IMOD software package [14]. Tilt series were aligned using gold fiducials, corrected using contrast transfer function (CTF), and dose-filtration implemented in IMOD. Reconstruction was performed by weighted back-projections with a simultaneous iterative reconstruction technique (SIRT)-like filter equivalent to 5 iterations. For visualization, 10 slices of the final tomogram were averaged.

**Indirect immunofluorescence analysis**

HuH7 cells were seeded on cover slips and infected with the respective viruses under BSL4 conditions with an MOI of 0.3. Cells were fixed after 24 h by 4% PFA treatment and cover slips were extracted from the BSL4 facility in 4% PFA after inactivation according to our regulations. For the detection of recombinantly expressed NP, HuH7 cells were transfected with 500 ng pCAGGS expression plasmid coding for the indicated NP mutant. Cells were fixed in 4% PFA 24 hpt. Indirect immunofluorescence staining was performed as described elsewhere [16]. Briefly, the PFA fixated cells were permeabilized with 0.1% Triton for 10 min, aldehyde groups were masked with 100 mM Glycine for 10 min. Subsequently, unspecific binding was blocked with blocking buffer (2% BSA, 5% Glycerin, 0.1% Tween20 and 0.05% NaN_3_ in PBS) for 10 min and primary antibodies (anti-NP from chicken and anti-VP40 from mouse) were diluted 1:50 in blocking buffer and incubated for 1 h. Cells were washed twice with PBS and incubated for 1 h in secondary antibody (anti-chicken IgG Alexa 488 (Dylight abcam, ab96947), anti-mouse IgG Alexa 594 (Thermo Fisher, A11005) diluted 1:400 in blocking buffer and DAPI 1:2000. Finally, cells were washed three times in PBS, mounted in fluor save reagent (Merck Millipore, 345789) and imaged using a Stellaris confocal scanning laser microscope from Leica with a 63x objective. Images were processed with ImageJ/Fiji.

**Mass spectrometry**

Viruses were grown on HuH7 cells and viral particles were purified from the supernatant as described for cryo-EM analysis. Inactivation was achieved by cooking samples in at least 1 % SDS two times for 10 min at 100 °C each.

A portion of pelleted viral particles corresponding to 25 µg of protein was transferred to a fresh tube and diluted to 40 µl. For the reduction of disulphide bridges 5 mM TCEP was added. Samples were then incubated for 15 min at 90°C. Subsequently, the resulting sulfhydryl-groups were chemically modified by adding iodoacetamide to a final concentration of 10 mM and incubating samples for 30 minutes at room temperature (RT) in the dark. 4 µl SP3-bead slurry [17] and 50 µl Acetonitrile (ACN) were added. Tubes were quickly vortexed, and incubated for 15 min. Subsequently, beads were separated using a magnetic separator and supernatant was discarded. 500 µl 70% Ethanol was added, beads agitated and subsequently separated. Supernatant was discarded and ethanol wash was repeated. 200 µl ACN were added, tubes were agitated and after magnetic separation, supernatant was discarded. Beads were dried. 100 µl digestion buffer (10% ACN/50 mM Ammoniumbicarbonate) containing trypsin was added to the dried beads. After resuspending the beads, samples were incubated in a thermomixer overnight at 30°C at 1200 rpm. Beads were separated using a magnet and supernatant was transferred into new collection-tubes. 30 µl of 2% DMSO were added to the beads and samples were sonicated for 5 min in an ultrasonic bath. Following, beads were separated and supernatant was transferred to the corresponding collection tube. Then, 30 µl of water (HPLC grade) was added to the beads, samples were vortexed. After quickly spinning the liquid down, beads were separated on a magnet and supernatant was transferred into the corresponding collection-tube. Combined elution solutions were acidified by the addition of 10 µl of 5% TFA. Reduced and alkylated peptides were then desalted and concentrated using Chromabond C18WP spin columns (Macherey-Nagel, Part No. 730522) according to manufacturer protocols. Finally, peptides were dissolved in 25 µl of water with 5% acetonitrile and 0.1% formic acid. A portion was diluted to a concentration of approximately 100 ng of peptides per microliter. The mass spectrometric analysis of the samples was performed using a timsTOF Pro mass spectrometer (Bruker Daltonic). A nanoElute HPLC system (Bruker Daltonics), equipped with an Aurora C18 RP column (25cm x 75µm ID) filled with 1.7 µm beads (IonOpticks, Australia) was connected online to the mass spectrometer. A portion of approximately 200 ng of peptides corresponding to 2 µl was injected directly on the separation column. Sample Loading was performed at a constant pressure of 800 bar. Separation of the tryptic peptides was achieved at 60°C column temperature with the following gradient of water/0.1% formic acid (solvent A) and acetonitrile/0.1% formic acid (solvent B) at a flow rate of 400 nl/min: Linear increase from 2%B to 17%B within 18 minutes, followed by a linear gradient to 25%B within 9 min and linear increase to 37% solvent B in additional 3 min. Finally, B was increased to 95% within 10 min and hold at 95% for additional 10 min. The built-in “DDA PASEF-standard_1.1sec_cycletime” method developed by Bruker Daltonics was used for mass spectrometric measurement.

Protein data analysis was performed using MaxQuant (Version 2.3.0.0). Measurements were done as technical duplicates, and the mean intensities of the replicates were further analysed. Search for protein phosphorylation was done on the same mass spec raw data using Proteome Discoverer 2.4 (ThermoScientific) or MaxQuant (Version 2.5.1.0). Phosphorylation sites in NPwt were classified as valid if they were reliably detected by both analysis algorithms (Proteome Discoverer and MaxQuant) in at least two replicates (technical or biological). The stacked bar figure (Figure 2G) was created with Graphpad Prism (version 10.0.2), based on log2 intensities of identified viral proteins (Supplement S4).

Data availability: The mass spectrometry proteomics data have been deposited to the ProteomeXchange Consortium via the PRIDE [18] partner repository with the dataset identifier PXD056320 (Reviewer account: Username: [reviewer_pxd056320@ebi.ac.uk](https://home.staff.uni-marburg.de/imp/dynamic.php?page=mailbox), password: rcdsRTVbdY1V).

References

[1] Biedenkopf N, Lier C, Becker S. Dynamic Phosphorylation of VP30 Is Essential for Ebola Virus Life Cycle. J Virol. 2016;90:4914–4925.

[2] Lier C, Becker S, Biedenkopf N. Dynamic phosphorylation of Ebola virus VP30 in NP-induced inclusion bodies. Virology. 2017;512:39–47.

[3] Pauly D, Chacana PA, Calzado EG, et al. IgY technology: extraction of chicken antibodies from egg yolk by polyethylene glycol (PEG) precipitation. J Vis Exp. 2011. DOI: 10.3791/3084.

[4] Rohde C, Pfeiffer S, Baumgart S, et al. Ebola Virus Activates IRE1α-Dependent XBP1u Splicing. Viruses. 2022;15. DOI: 10.3390/v15010122.

[5] Hoenen T, Groseth A, Kolesnikova L, et al. Infection of naive target cells with virus-like particles: implications for the function of ebola virus VP24. J Virol. 2006;80:7260–7264.

[6] Watt A, Moukambi F, Banadyga L, et al. A novel life cycle modeling system for Ebola virus shows a genome length-dependent role of VP24 in virus infectivity. J Virol. 2014;88:10511–10524.

[7] Hoenen T, Jung S, Herwig A, et al. Both matrix proteins of Ebola virus contribute to the regulation of viral genome replication and transcription. Virology. 2010;403:56–66.

[8] Creytz I von, Gerresheim GK, Lier C, et al. Rescue and characterization of the first West African Marburg virus 2021 from Guinea. Heliyon. 2023;9:e19613.

[9] Krähling V, Dolnik O, Kolesnikova L, et al. Establishment of fruit bat cells (Rousettus aegyptiacus) as a model system for the investigation of filoviral infection. PLoS Negl Trop Dis. 2010;4:e802.

[10] Hierholzer JC, Killington RA. Virus isolation and quantitation. In; p. 25–46.

[11] Kruse T, Biedenkopf N, Hertz EPT, et al. The Ebola Virus Nucleoprotein Recruits the Host PP2A-B56 Phosphatase to Activate Transcriptional Support Activity of VP30. Mol Cell. 2018;69:136-145.e6.

[12] Bach S, Demper J-C, Klemm P, et al. Identification and characterization of short leader and trailer RNAs synthesized by the Ebola virus RNA polymerase. PLoS Pathog. 2021;17:e1010002.

[13] Winter SL, Golani G, Lolicato F, et al. The Ebola virus VP40 matrix layer undergoes endosomal disassembly essential for membrane fusion. EMBO J. 2023;42:e113578.

[14] Mastronarde DN. Automated electron microscope tomography using robust prediction of specimen movements. J Struct Biol. 2005;152:36–51.

[15] Hagen WJH, Wan W, Briggs JAG. Implementation of a cryo-electron tomography tilt-scheme optimized for high resolution subtomogram averaging. J Struct Biol. 2017;197:191–198.

[16] Kolesnikova L, Berghöfer B, Bamberg S, et al. Multivesicular bodies as a platform for formation of the Marburg virus envelope. J Virol. 2004;78:12277–12287.

[17] Hughes CS, Moggridge S, Müller T, et al. Single-pot, solid-phase-enhanced sample preparation for proteomics experiments. Nat Protoc. 2019;14:68–85.

[18] Perez-Riverol Y, Bai J, Bandla C, et al. The PRIDE database resources in 2022: a hub for mass spectrometry-based proteomics evidences. Nucleic Acids Res. 2022;50:D543-D552.
